# Supplementary figures and images for: Recognition of commensal bacterial peptidoglycans defines Drosophila gut homeostasis and lifespan
Source: PLoS Genet. 2023 Apr 6;19(4):e1010709. doi: 10.1371/journal.pgen.1010709 (PMC10112789; doi:10.1371/journal.pgen.1010709)

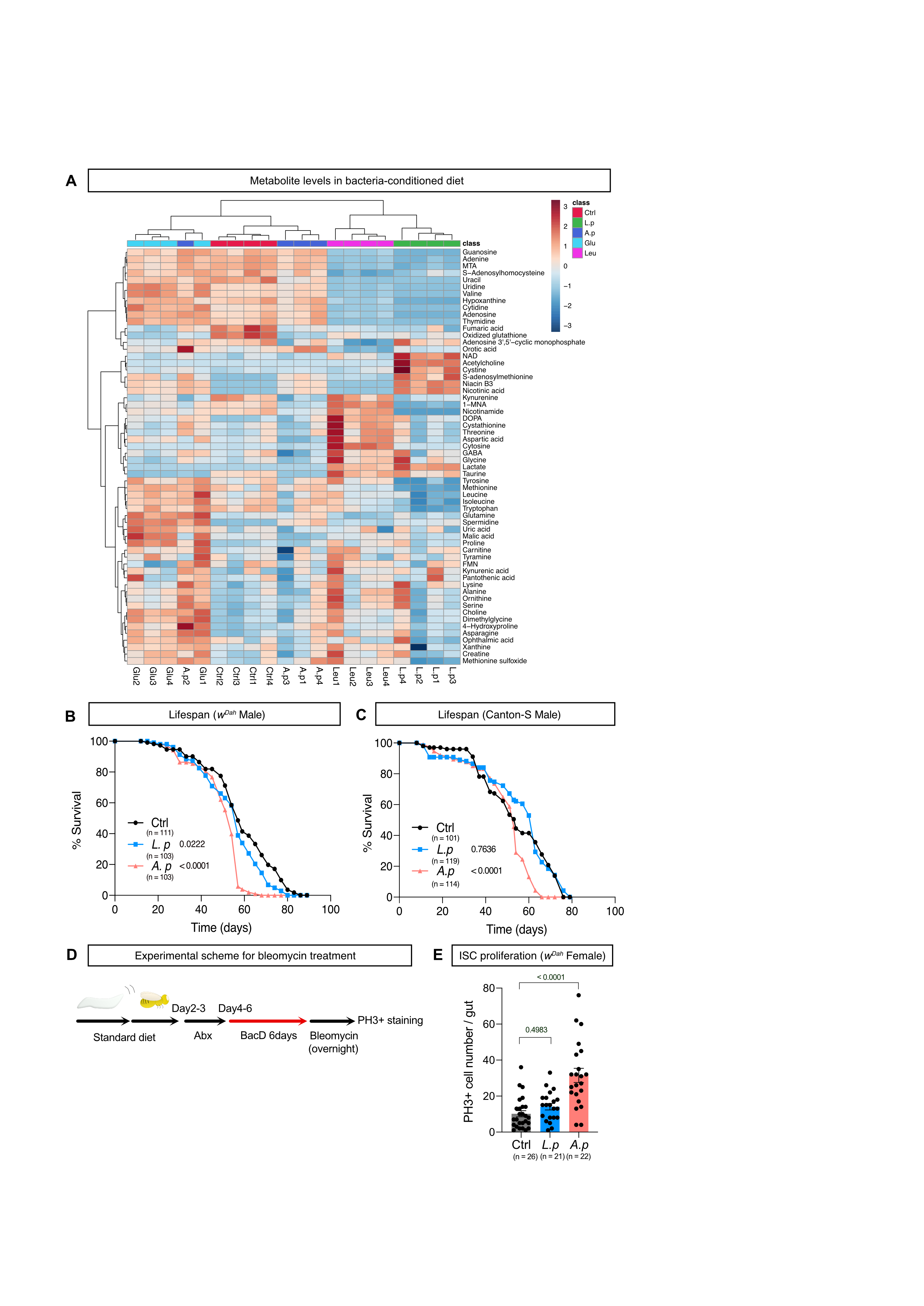

Supplement: S1 Fig — (A) The effect of bacterial conditioning on metabolite levels. (B)(C) Lifespan of male wDah (B) and Canton-S (C) flies with BacD. A log-rank test was used to compare between control (Ctrl) and each BacD. (D) Experimental scheme of bleomycin treatment in the phospho-histone H3-positive cell number counting experiment. (E) Phospho-histone H3-positive cell numbers in the midgut of wDah female flies after five days of BacD and overnight treatment with 2.5 μg/mL bleomycin. The control diet followed the same procedure for BacD but it has only MRS broth in place of bacterial isolates, resulting in the antibiotics-contained diet. For the statistics, one-way ANOVA with Holm-Šídák’s multiple comparison was used. Sample sizes (n) and P values are in each figure. (TIFF) [file pgen.1010709.s001.tiff]

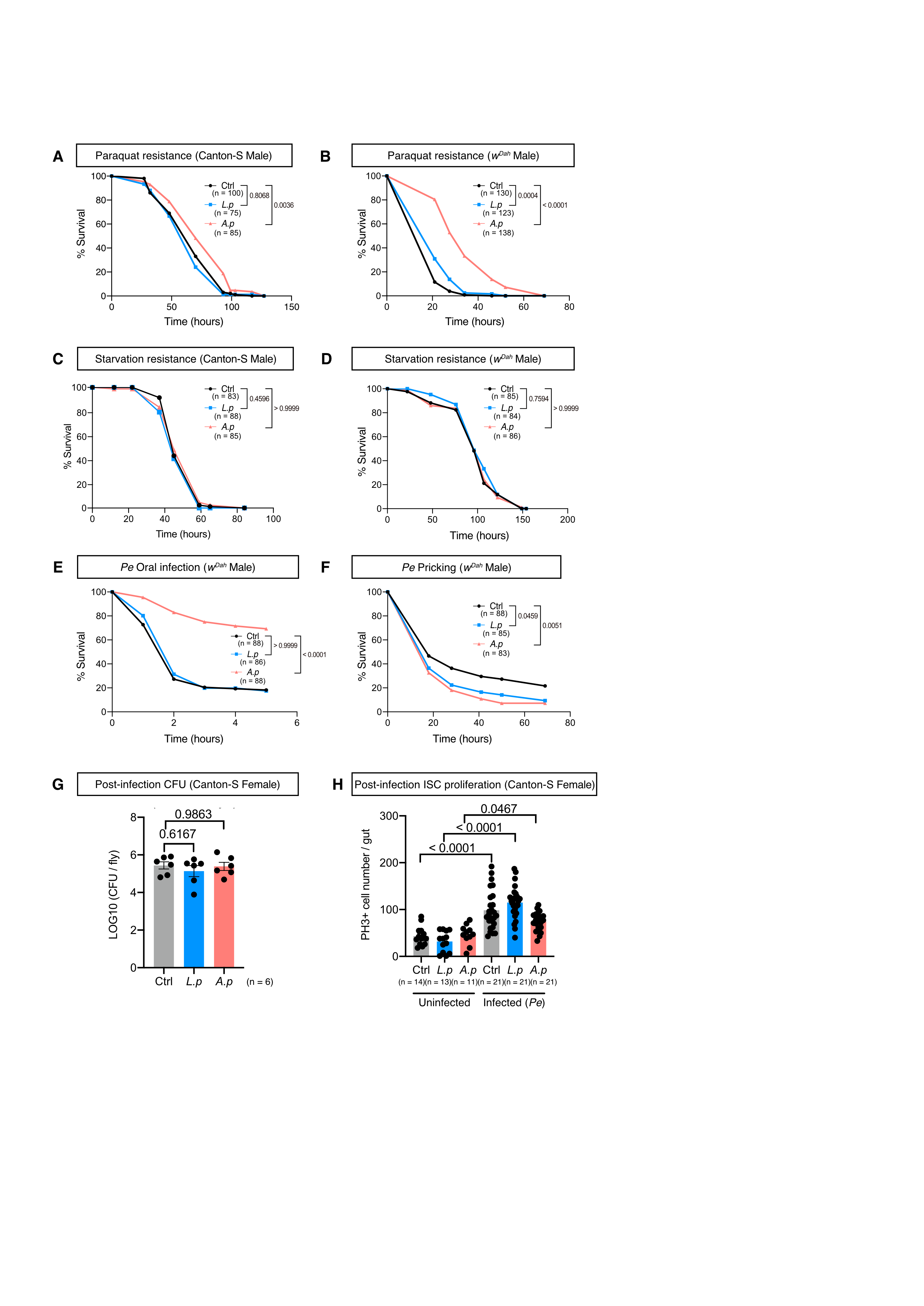

Supplement: S2 Fig — (A)(B) Survival curve of male Canton-S (A) and wDah (B) flies during 10 mM paraquat feeding after 5 days of a bacteria-conditioned diet (BacD). A log-rank test was used to compare between control (Ctrl) and each BacD. (C)(D) Survival curve of male Canton-S (C) and wDah (D) flies during starvation stress after 5 days of BacD. A log-rank test was used to compare between control (Ctrl) and each BacD. (E)(F) Survival curve of male Canton-S flies orally (E) or septically (F) infected with Pseudomonas entomophila (Pe) after 5 days of BacD. A log-rank test was used to compare between control (Ctrl) and each BacD. (G)(H) Colony forming units (CFUs) (G) and phospho-histone H3-positive cell numbers (H) in the midgut of female after 6 (G) or 16 (H) hours after oral infection with P.entomophila. Canton-S flies used were given BacD for 5 days before the assay. For the statistics, One-way ANOVA with Holm-Šídák’s multiple comparison was used. The control diet followed the same procedure for BacD but it has only MRS broth in place of bacterial isolates, resulting in the antibiotics-contained diet. Sample sizes (n) and P values are in each figure. (TIFF) [file pgen.1010709.s002.tiff]

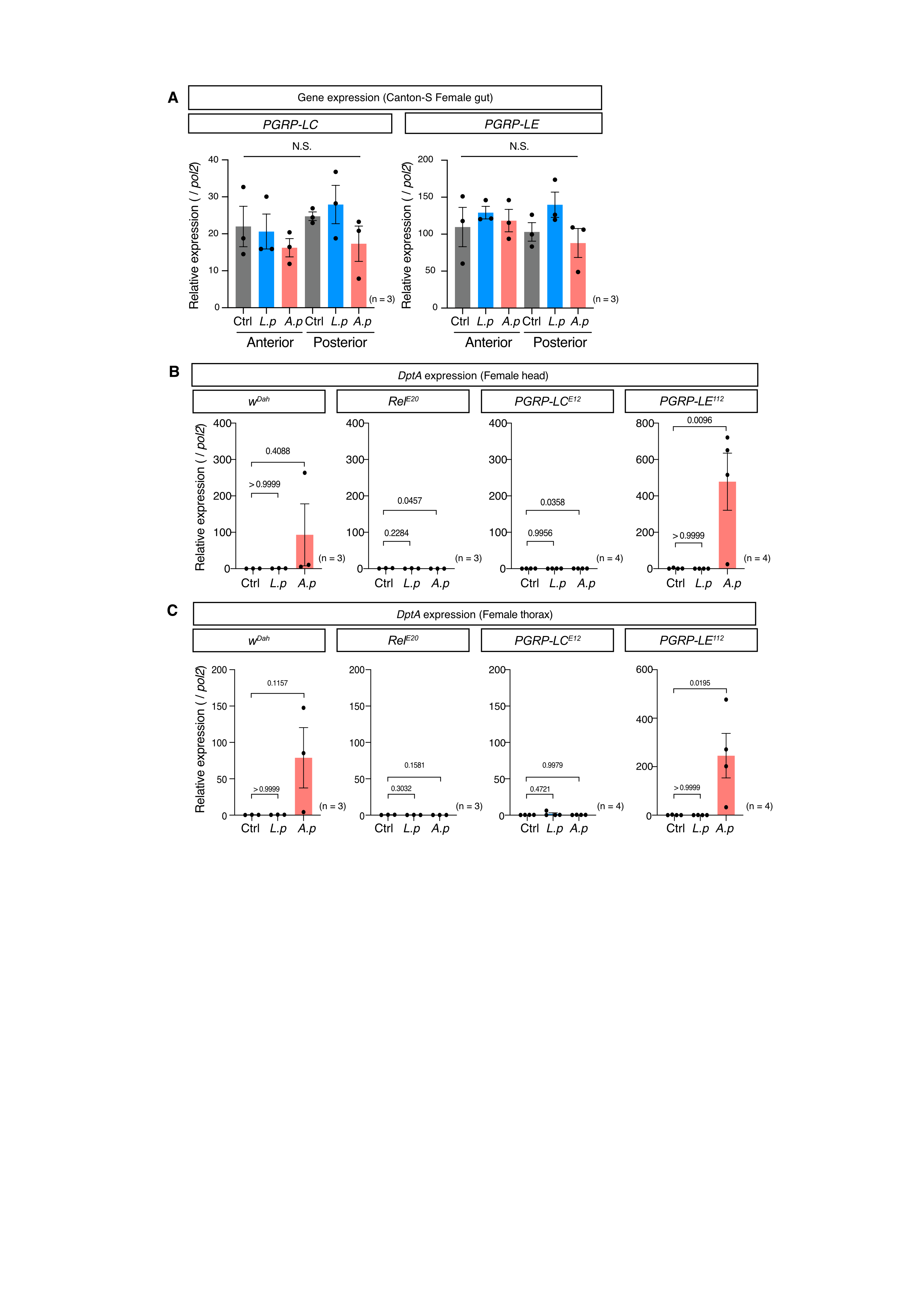

Supplement: S3 Fig — (A) Quantitative RT–PCR of the genes PGRP-LC and PGRP-LE in female Canton-S anterior and posterior gut after 24 hours of BacD. One-way ANOVA with Holm-Šídák’s multiple comparison was used. (B, C) Quantitative RT–PCR of DptA in female wDah, RelE20, PGRP-LCE12, and PGRP-LE112 fly heads (B) and thoraxes (C) after 24 hours of BacD. One-way ANOVA with Holm-Šídák’s multiple comparison was used. The control diet followed the same procedure for BacD but it has only MRS broth in place of bacterial isolates, resulted in the antibiotics-contained diet. Sample sizes (n) and P values are in each figure. (TIFF) [file pgen.1010709.s003.tiff]

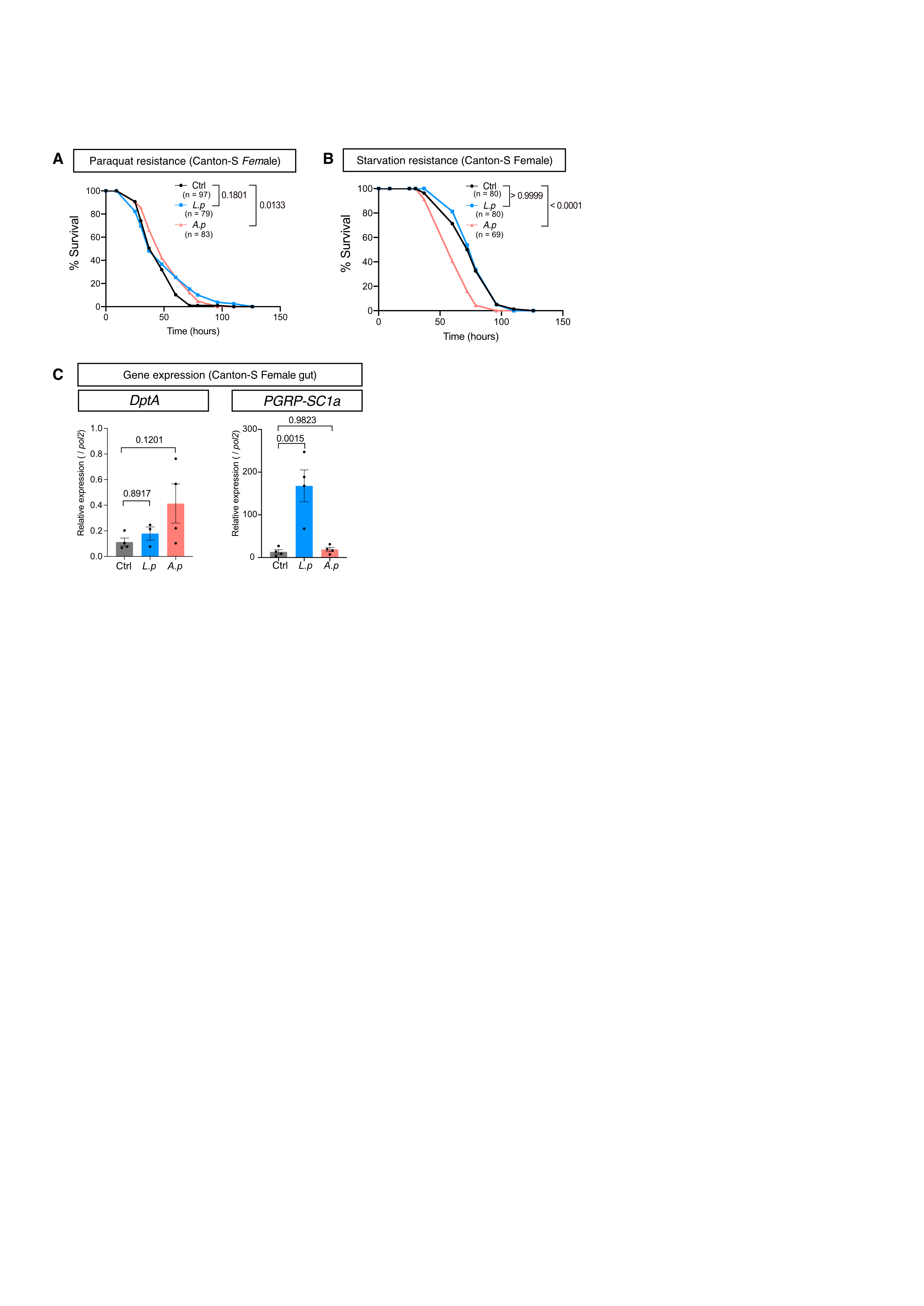

Supplement: S4 Fig — (A) Survival curve of female Canton-S flies during 10 mM paraquat feeding after 5 days of a bacteria-conditioned diet (BacD) without adding the antibiotics cocktail. A log-rank test was used to compare between control (Ctrl) and BacD without antibiotics. (B) Survival curve of female Canton-S flies during starvation stress after 5 days of BacD without adding the antibiotics cocktail. A log-rank test was used to compare between control (Ctrl) and BacD without antibiotics. (C) Quantitative RT–PCR of DptA in female Canton-S fly gut after 24 hours of BacD without adding the antibiotics cocktail. These experiments were conducted following the exact same scheme as BacD experiments, except that the BacD was prepared without the addition of the antibiotics cocktail. For the statistics, one-way ANOVA with Holm-Šídák’s multiple comparison was used. The control diet has only MRS broth in place of bacterial isolates without the antibiotics cocktail. Sample sizes (n) and P values are in each figure. (TIFF) [file pgen.1010709.s004.tiff]

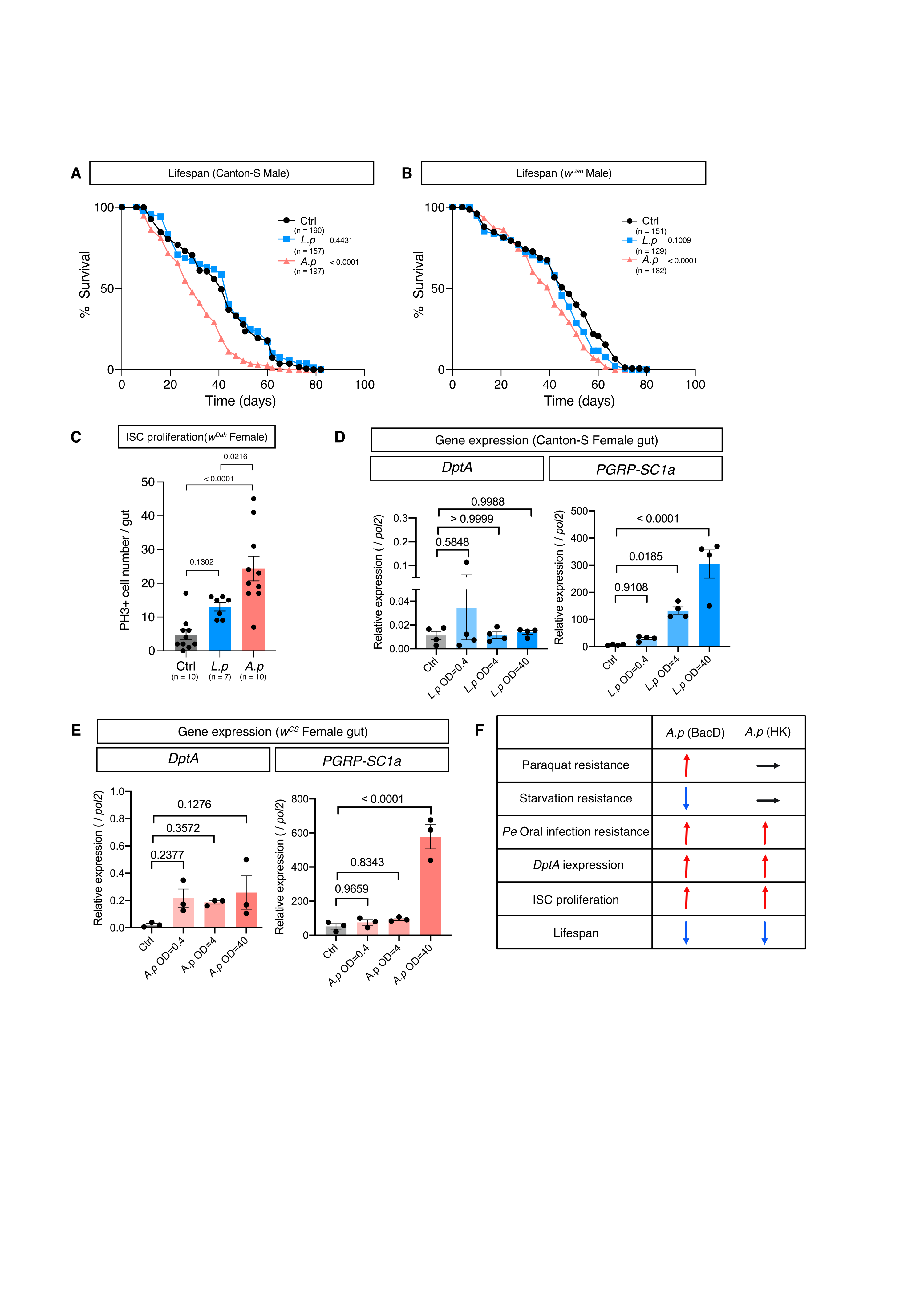

Supplement: S5 Fig — (A)(B) Lifespan of male Canton-S (A) and wDah (B) flies with the heat-killed (HK) diet. A log-rank test was used to compare between control (Ctrl) and each HK diet. (C) Phospho-histone H3-positive cell numbers in the midgut of female wDah flies fed the HK diet for 40 days. (D) (E) Quantitative RT–PCR of DptA and PGRP-SC1a after 24 hours of several dilutions of HK diet with either L.plantarum (D) or A.persici (E). For the statistics, one-way ANOVA with Holm-Šídák’s multiple comparison was used. (F) The summary of phenotypes in flies with BacD and HK diet. The control diet is the antibiotics-contained diet. Sample sizes (n) and P values are in each figure. (TIFF) [file pgen.1010709.s005.tiff]

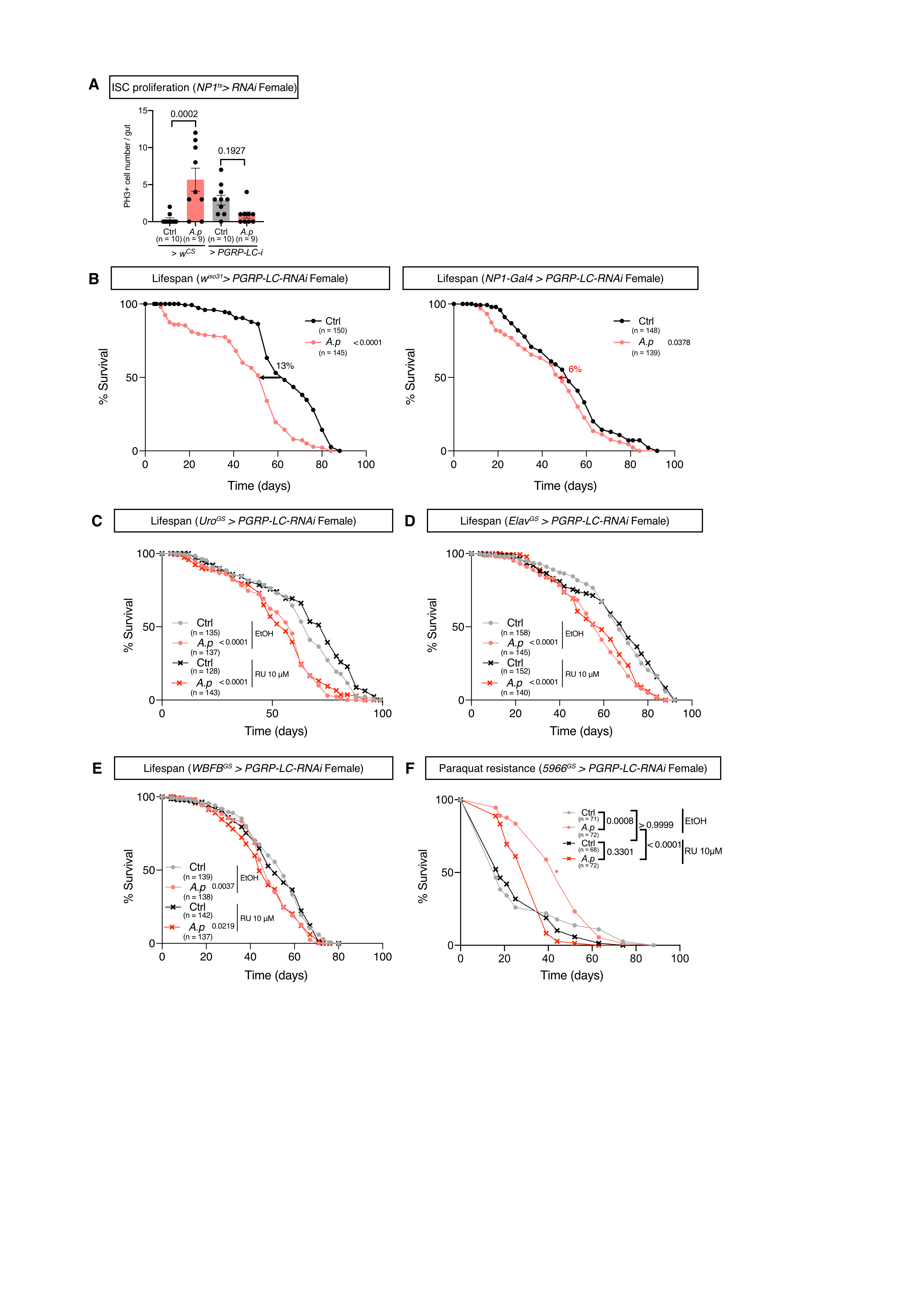

Supplement: S6 Fig — (A) Phospho-histone H3-positive cell numbers in the midgut of NP1ts > PGRP-LC-RNAi flies after 38days of heat-killed (HK) diet. Flies were reared at 30°C during their adult period. For the statistics, one-way ANOVA with Holm-Šídák’s multiple comparison was used. (B) Lifespan of female wiso31 > PGRP-LC-RNAi and NP1-Gal4 > PGRP-LC-RNAi flies with HK diet. The 50% survival rate is compared. (C)-(E) Lifespan of female UroGS, ElavGS, and WBFBGS > PGRP-LC-RNAi flies with HK diet and ethanol or 10 μM RU. (F) Survival curve of female flies during 10 mM paraquat feeding after 5 days of BacD and ethanol or RU 10μM. A log-rank test was used to compare between control (Ctrl) and BacD with ethanol or 10μM RU. The control diet in (A)-(E) is the antibiotics-contained diet. The control diet in (F) followed the same procedure for BacD but it has only MRS broth in place of bacterial isolates, resulted in the antibiotics-contained diet. Sample sizes (n) and P values are in each figure. (TIFF) [file pgen.1010709.s006.tiff]
