# Supplementary material for: Recognition of commensal bacterial peptidoglycans defines Drosophila gut homeostasis and lifespan
Source: PLoS Genet. 2023 Apr 6;19(4):e1010709. doi: 10.1371/journal.pgen.1010709 (PMC10112789; doi:10.1371/journal.pgen.1010709)
Supplement: Attachment — Submitted filename: Reviewer comments PlosGenet BacD-LC v9.pdf [file pgen.1010709.s010.pdf]

Reviewer #1: In this manuscript, Onuma and colleagues investigated how Acetobacter persici - a member of Drosophila melanogaster gut microbiota shortens the host lifespan. The authors used a bacteria-conditioned diet with antibiotics instead of alive bacteria to minimize contamination. Flies fed Acetobacter- but not Lactiplantibacillus-conditioned diet were short-lived, had increased intestinal stem cell proliferation and increased survival after intestinal infection with Pseudomonas entomophila. Transcriptomic analysis revealed that A. persici preferably induces antimicrobial peptides (AMPs),

while L. plantarum upregulates negative regulators of Imd pathway. Such differences in the induction of Imd target genes are likely due to differential activation of Imd pathway receptors, PGRP-LC and PGRP-LE by the two bacteria. This work suggests that differential ability of gut commensals to stimulate immune receptors results in distinct host response which determines the impact of gut bacteria on host lifespan. The authors performed an extensive comparative analysis to understand the differential impact of two gut commensals of the fruit fly lifespan. Additionally, they introduce a new technique - bacteria-conditioned diet to study microbial impact on host lifespan. Therefore, the manuscript is of interest to broad audience of Plos Genetics. However, certain issued still need to be resolved.

Comments:

1-1. The authors need to clearly state what control is in all their graphs.

> We added a description of the control group in each figure legend as follows.

(Figure1-5, S1-S3 legends) The control diet followed the same procedure for BacD but it has only MRS broth in place of bacterial isolates, resulting in the antibiotics-contained diet.

(Figure6-8, S4-S5 legends) The control diet is the antibiotics-contained diet.

1-2. For bacteria-conditioned medium it is claimed that only the culture medium was used. Was this medium treated with antibiotics similar to bacteria-inoculated food? This is an important point, since antibiotics might themselves affect the host lifespan.

>Antibiotics were added in all groups including the control (only MRS broth). We added this information in the Results and Methods section.

(Result) *The same antibiotic cocktail was added for the negative control to compare the effect of the bacterial components.*
(Methods) *Finally, 100-120 µL of antibiotic cocktail (rifampicin 10 g/L (Wako 185-01003), tetracycline 2.5 g/L (Wako 203-08592), and ampicillin 12.5 g/L (Wako 203-08592) in 100mM HCl) was added to each diet (including the negative control) to prevent further conditioning.*

1-3. Did the authors verify that their antibiotics cocktail actually kills all bacteria (Fig 1b)? This is essential to check (quantify viable bacteria after antibiotic treatment) since one possibility could be that Acetobacteriaceae are not killed by antibiotics and all the phenotypes that the authors observed are due to the presence or absence of alive bacteria.
>We have carefully determined the dosage of antibiotics based on the viability of bacteria in BacD diets. We periodically check the absence of live bacteria. We added this explanation in the Methods.

(Methods) *The dosage of antibiotics was optimised based on the viability of bacteria in BacD. The absence of live bacteria in BacD was periodically checked by plating a piece of BacD to MRS agar.*

1-4. Another import point to address is whether flies feed on such bacteria-conditioned diet especially after the addition of antibiotics. What if flies like Acetobacter-conditioned diet more then Lactobacillus-conditioned diet? They will eat more Acetobacter diet, therefore inducing stronger IMD response.
> We thank the reviewer for this great suggestion. To test whether the BacD affects flies' appetite, we performed the capillary feeder (CAFE) assay. Basically, we observed no differences in feeding behaviour. These data suggest that BacD would not influence the feeding behaviour, denying the possibility that the flies eat more of the *A. persici*-conditioned diet. We added this data in the revised manuscript.

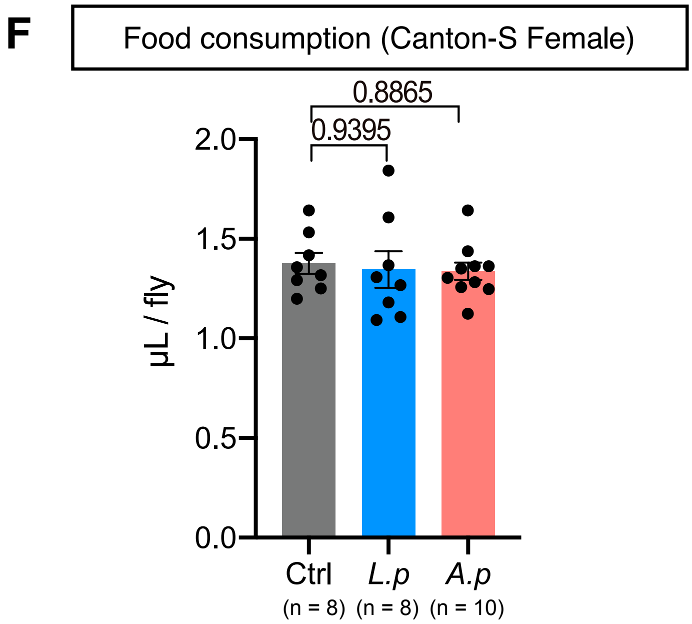

Fig. 2F, Food consumption of female flies by CAFE assay. Flies were treated with BacD for five days. Each point shows data of 7 or 8 flies. For the statistics, One-way ANOVA with Holm-Šídák's multiple comparison was used.

(Results) *Our data indicated that the feeding of BacD with A. persici Ai produced stronger phenotypes than that with L. plantarum Lsi. One hypothesis was that the flies consumed more A. persici Ai-conditioned diet because of the increased appetite, leading to a greater response. To evaluate this possibility, we conducted the capillary feeder assay (CAFE). This assay provides a measure of the flies' long-term feeding behaviour that quantifies the appetite of the flies. However, no differences were observed in feeding behaviour (Fig 2F). This finding suggests that BacD does not influence the behaviour, and therefore, the differential effect of each BacD on the phenotype of the flies cannot be explained by the amount of food intake.*

The result of CAFE assay showed how hungry flies are under BacD. In contrast, the blue-dye assay would quantify the short-term feeding behaviour that indicates how flies prefer to eat the diet. However, it is technically difficult to combine BacD and Blue dye, as we culture the bacteria in the solid fly diet. Instead, we used HK bacteria. As shown in Fig. R1, we again observed no differences in feeding behaviour using HK Blue

dye assay. As this experiment has been done only once, we did not add the data in the revised manuscript. However, it is suggested that there is little effect of HK on food consumption.

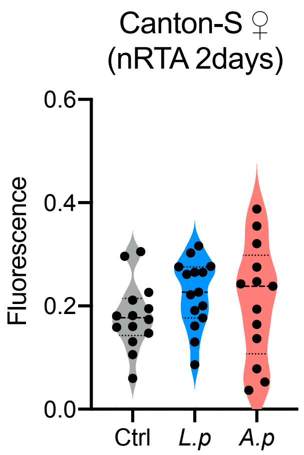

Fig. R1, Quantification of food consumption of female flies by blue dye assay. Antibiotics-treated flies were fed with HK diet with blue dye. Each point shows data for a single fly.

1-5. Fig 2E. Time should be days not hours.

> We thank the reviewer for pointing this out. This figure was corrected in the revised manuscript.

1-6. The authors claim: "it is possible that increased ISC proliferation in flies fed with the *A. persici* Ai-conditioned diet would be beneficial for the acceleration of the damaged epithelia but detrimental for maintaining healthy tissue during ageing." To provide further support for this claim ph3 counts should be performed after Pe infection. Also, we do not know if Acetobacter promotes resistance or tolerance to Pe infection. If, as authors suggest, flies survive better because they have increased gut repair - this rather fulfills definition of tolerance. However, if it is due to stronger IMD activity and pathogen killing - it is resistance. Quantification of PE load will help to resolve this issue.

>We thank the reviewer for asking this critical question. As requested, we performed PH3 count and quantification of PE load upon PE infection. We found that CFU count

was not affected by BacD, suggesting that resistance is not changed. To our surprise, the number of ISC proliferation in flies fed a A. persici-conditioned diet upon Pe infection was even decreased. This observation implied that A. persici-conditioned diet mitigates the gut damage and that A. persici improves tolerance. We added these data and the text in the revised manuscript.

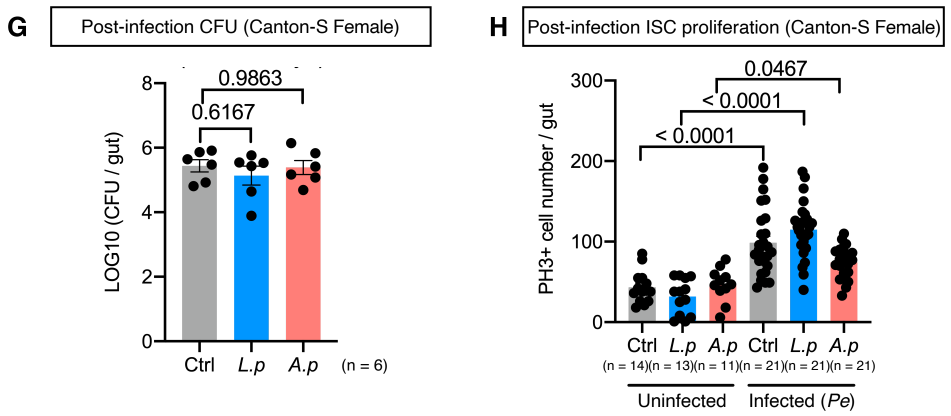

Fig S2G, H, Colony forming units (CFUs) (G)and phospho-histone H3-positive cell numbers (H) in the midgut of female after 6 (G) or 16 (H) hours after oral infection with *P.entomophila.* Canton-S flies used were given BacD for 5 days before the assay. For the statistics, One-way ANOVA with Holm-Šídák's multiple comparison was used.

(Result) To distinguish whether increased survival against P. entomophila represents increased tolerance or resistance, we performed quantifications of CFUs and ISC proliferation following oral infection with P. entomophila. The data showed that there was no decrease in CFUs or increase in ISC proliferation in flies fed a diet conditioned with A. persici Ai (Figs S2G, H). Instead, we observed that these flies displayed a relatively mild level of ISC proliferation compared to other groups (Fig S2H), which might imply a milder epithelial damage. These results suggest that the increased survival against P. entomophila observed in flies with A. persici Ai-conditioned diet may be due to increased tolerance rather than resistance to pathogen infection.

1-7. Better annotation of RNAseq table would be good. What does LvsM mean and the others? Did the authors found anything else in the RNAseq besides AMPs and PGRPs?

>We appologise for not clarifying what the legends mean. Basically, L stands for *Lactiplantibacillus plantarum* Lsi, A for *Acetobacter persici* Ai, and M for MRS broth-only negative control. Thus, LvsM tab in the supplementary Tables shows the list of genes differentially expressed in the female gut from *L. plantarum* Lsi-conditioned diet compared to that from the negative control. We have clarified it in each tab. Also, we added a brief description of what genes and pathways are altered in each condition in the revised manuscript.

(Result) The Gene Ontology (GO) analysis of downregulated genes revealed that "Proteolysis" was significantly altered (FDR<0.01) in both A. persici Ai and L. plantarum Lsi (Supplementary Table 2), suggesting a potential role in the digestion of food. In contrast, the GO analysis of upregulated genes by A. persici Ai showed highly significant pathways related to carbohydrate metabolic processes such as glycolytic or pentose-phosphate pathway, as well as innate immune response or defense response. "Determination of adult lifespan" was found only in A. persici Ai (Supplementary Table 2). Interestingly, the GO analysis of upregulated genes in L. plantarum Lsi revealed that only two GO terms were below FDR<0.01 and these were related to amidase PGRPs (Supplementary Table S2).

1-8. "To elucidate how A. persici Ai, but not L. plantarum Lsi, shortens the lifespan and alters the host defence, we investigated the transcriptomic response of the gut to each BacD." Better phrasing needed because this sounds like the authors will do RNAseq of aging animals, which is not what they have done.
>We thank the reviewer for pointing this out. In the revised manuscript, we have rephased this sentence as follows.
(Result) To elucidate how A. persici Ai and L. plantarum Lsi distinctively impact the host, we investigated the transcriptomic response of the gut to each BacD.

1-9. "Interestingly, L. plantarum Lsi was also capable of inducing DptA from the posterior midgut, although it did not reach statistical significance due to the large

induction of DptA by A. persici Ai (Fig 4C)." How does large induction of DptA by A. persici affect statistical significance of DptA induction by L. plantarum?

> We deleted "due to the large induction of DptA by *A. persici* Ai".

1-10. "Therefore, we simply fed the flies heat-killed bacteria (Fig 6A)." Heat-killed bacteria still contain other things besides cell wall components. Therefore, attributing all the phenotypes with heat-killed bacteria to cell wall only or peptidoglycan is not correct. To make such claims, purified peptidoglycan has to be used.

>We thank the reviewer for this critical suggestion. During revision, we endeavored to purify peptidoglycans from both *A. persici* Ai and *L. plantarum* Lsi, using a published protocol (Schaub et al., *Bio Protoc.,* 2017). Since total amount of PGNs from gram negative *A. persici* Ai and gram positive *L. plantarum* Lsi are very different, we measured OD_{254} to quantify PGN concentration (according to the protocol) and fed the female flies with the equal amount of PGNs. Strikingly, we observed that feeding PGNs phenocopied BacD or HK for Imd activation patterns in the gut. Purified PGNs of *A.persici* Ai induced both *DptA* and *PGRP-SC1a* expression, while PGNs from *L. plantarum* Lsi induced only *PGRP-SC1a* expression. Removing WTA of *L. plantarum* Lsi by HCl did not affect the gene expression pattern at all. Since we use a relatively high amount of bacteria culture (equivalent to OD_{600}=2000) to purify PGNs, we diluted PGNs. A hundred-fold dilution of purified PGNs of *A.persici* Ai did not diminish the activity to induce *DptA* expression. These data together suggest that purified PGNs are sufficient for the distinct stimulation of PGRP-LC/LE by two bacterial species. We added these data and discussion in the revised manuscript. Due to the difficulty in obtaining a large amount of PGN and the limited time for revision, we measured only expression levels of the Imd target genes in the gut as a primary response of flies. However, there are some studies suggesting the relevance of peptidoglycans solely based on the fact that they are dependent on PGRPs (Charroux et al., *PLOS Genetics,* 2022, Zugasti et al., *Scientific Reports*, 2020). Given that ageing phenotypes are mediated by a "peptidoglycan"-recognition protein PGRP-LC, and caused by heat-killed *A. persici* Ai, we argue that we can logically attribute these phenotypes to the PGNs. Further biochemical and genetic analyses are needed to understand how specific PGN structure is recognized by the two PGRP receptors.

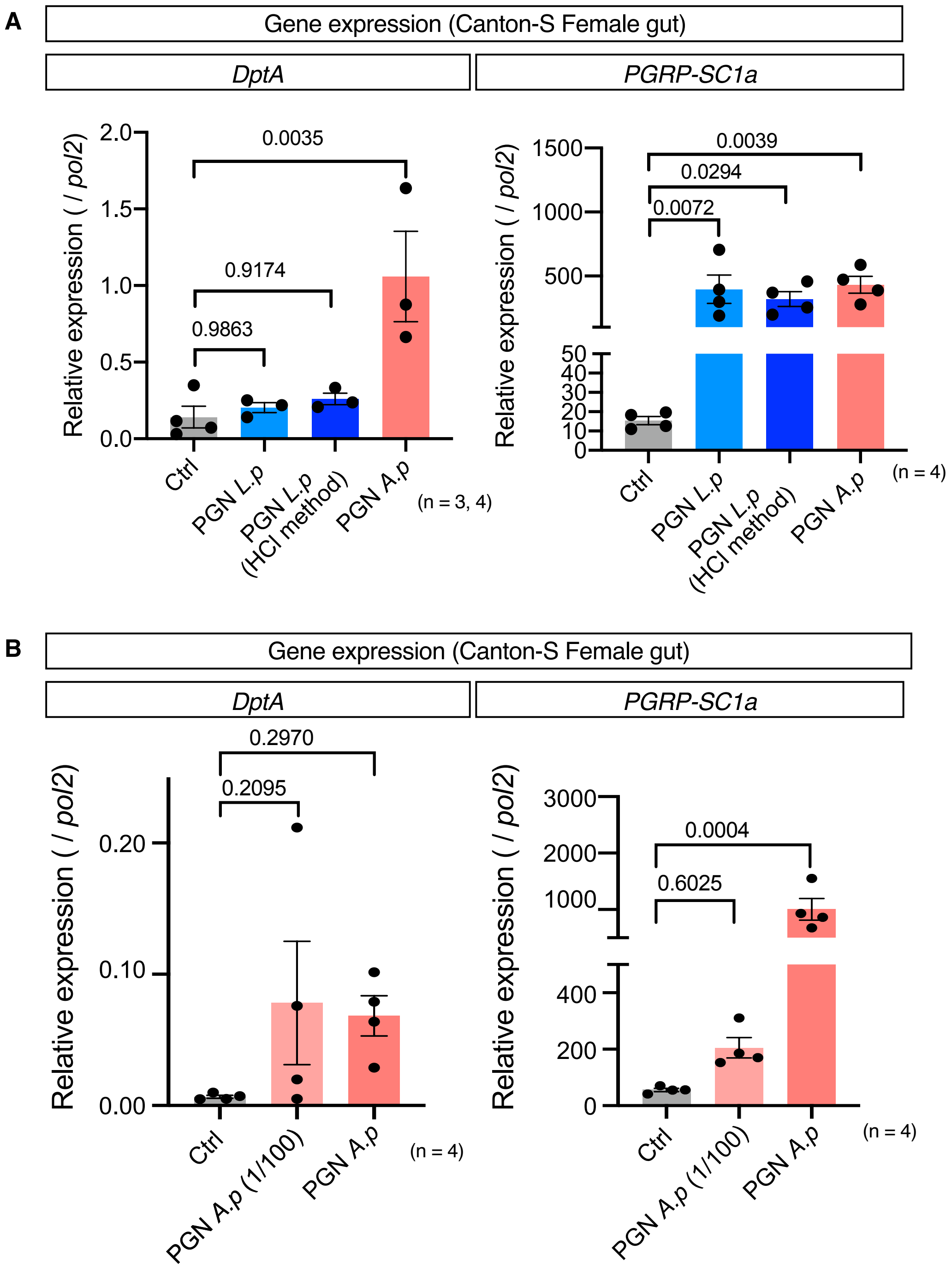

Fig 7. Feeding *L.plantarum* Lsi and *A.persici* Ai purified peptidoglycans.

(A)Quantitative RT–PCR of the genes *DptA* and *PGRP-SC1a* in female fly Canton-S guts after 24 hours of purified peptidoglycan diet with the same concentration (measured and calculated by OD_{254}). For the statistics, ANOVA with Holm-Šídák's multiple comparison was used. (B)Quantitative RT–PCR of the genes *DptA* and *PGRP-SC1a* in female fly

Canton-S guts after 24 hours of *A.persici* Ai purified peptidoglycan (1 or 100 times dilution compared to that used in (A)) diet. For the statistics, ANOVA with Holm-Šídák's multiple comparison was used. The control diet is the antibiotics-contained diet. Sample sizes (n) and *P* values are in each figure.

(Result)

To determine whether the differences in activity between heat-killed A. persici Ai and L. plantarum Lsi were solely attributed to the differences in PGNs rather than other components, we purified the PGNs from each bacterial species. As gram positive L. plantarum has wall teichoic acids (WTA) in the PGN layers, we also tested the effect of removing WTA on the phenotype. Strikingly, feeding with purified PGNs produced the same pattern of Imd target gene induction in the gut as BacD or HK (Fig 7A). Purified PGNs of A.persici Ai induced both DptA and PGRP-SC1a expression, while PGNs from L. plantarum Lsi induced only PGRP-SC1a expression in the gut. Removing WTA by HCl did not affect the gene expression pattern of L. plantarum Lsi at all. Furthermore, even a hundred-fold dilution of purified PGNs of A.persici Ai did not diminish the induction of DptA expression (Fig 7B). These results suggest that purified PGNs are sufficient for inducing the distinct gene expression patterns in the gut and the differences in PGN structure of two bacterial species are likely responsible for the differential stimulation of the receptors PGRP-LC/LE.

(Discussion) *Due to the difficulty in obtaining a large amount of purified PGN, we measured only expression levels of the Imd target genes in the gut as a primary response of flies. However, given that ageing phenotypes are mediated by a "peptidoglycan"-recognition protein PGRP-LC, and caused by heat-killed A. persici Ai, we can logically attribute these phenotypes to the PGNs. Further biochemical and genetic analyses are needed to understand how specific PGN structure is recognized by the two PGRP receptors.*

1-11. Methods "Septic and oral infection of Pseudomonas entomophila" Please specify

the dose of bacteria used for pricking and oral infection. There is typo in P. entomophila.

> We corrected the typo and specify the dose of bacteria (OD_{600} = 200) in the Methods.

(Methods) *The microbe solution was obtained by mixing a pellet of P. entomophila with a culture supernatant (1:1), resulting in the concentration of the bacteria was approximately OD_{600} = 200.*

Reviewer #2: Using Drosophila as a model, Onuma at al investigate the impact of bacteria-conditioned diet and heat-killed bacteria on several host physiological traits: lifespan, intestinal stem cell proliferation, infection, resistance to oxidative stress and activation of host defenses through IMD pathway. The authors identified a trade-off between lifespan and host defenses in presence of A. persici conditioned diets. Although conceptually interesting, this work merely reports a set of initial interesting observations that deserve further analysis but at this stage the work raise several questions regarding the experimental designs.

2-1, The interesting observations that bacteria-conditioned diets influence fly lifespan differently given the bacteria used are currently embedded with a lot of experimental testing of already established facts about how Acetobacter spp and Lactobacillacae strains regulates AMPs and PGRPs at the transcriptional level in the adult fly gut. One major concern refers to the use of 'peptidoglycan' to refer to 'heat-killed bacteria' throughout the manuscript including the title. It's misleading and incorrect. To address this issue, I would suggest to extract peptidoglycan from the implicated strains, determine their composition (and structure) and test for the claimed phenotypes.
>As stated in our comment 1-10, during revision, we endeavored to purify peptidoglycans from both *A. persici* Ai and *L. plantarum* Lsi, using a published protocol (Schaub et al., *Bio Protoc.,* 2017). Since total amount of PGNs from gram negative *A. persici* Ai and gram positive *L. plantarum* Lsi are very different, we measured OD_{254} to quantify PGN concentration (according to the protocol) and fed the female flies with the equal amount of PGNs. Strikingly, we observed that feeding PGNs phenocopied BacD or HK for Imd activation patterns in the gut. Purified PGNs of *A.persici* Ai induced both *DptA* and *PGRP-SC1a* expression, while PGNs from *L. plantarum* Lsi induced only *PGRP-SC1a* expression. Removing WTA of *L. plantarum* Lsi by HCl did not affect the gene expression pattern at all. Since we use a relatively high amount of bacteria culture (equivalent to OD_{600}=2000) to purify PGNs, we diluted PGNs. A hundred-fold dilution of purified PGNs of *A.persici* Ai did not diminish the activity to induce *DptA* expression. These data together suggest that purified PGNs are sufficient for the distinct stimulation of PGRP-LC/LE by two bacterial species. We added these data and discussion in the revised manuscript. Due to the difficulty in obtaining a large amount of PGN and the limited time for revision, we measured only expression levels of the

Imd target genes in the gut as a primary response of flies. However, there are some studies suggesting the relevance of peptidoglycans solely based on the fact that they are dependent on PGRPs (Charroux et al., *PLOS Genetics,* 2022, Zugasti et al., *Scientific Reports*, 2020). Given that ageing phenotypes are mediated by a "peptidoglycan"-recognition protein PGRP-LC, and caused by heat-killed *A. persici* Ai, we argue that we can logically attribute these phenotypes to the PGNs. We thank the reviewer for suggesting this striking experiment. These new data have solidified our conclusion.

We think determining the composition and structure of PGNs is neither feasible nor mandatory at this stage. To elucidate how PGN is involved, specific analytical and synthetic chemistry with genetic and biochemical analyses are required. In addition, deep mechanistic investigations to reveal how PGNs from each bacterium differ in the capacity to bind different PGRPs *in vitro* and *in vivo* from the view point of structural biology are necessary. We believe it is reasonably beyond the scope of the present study.

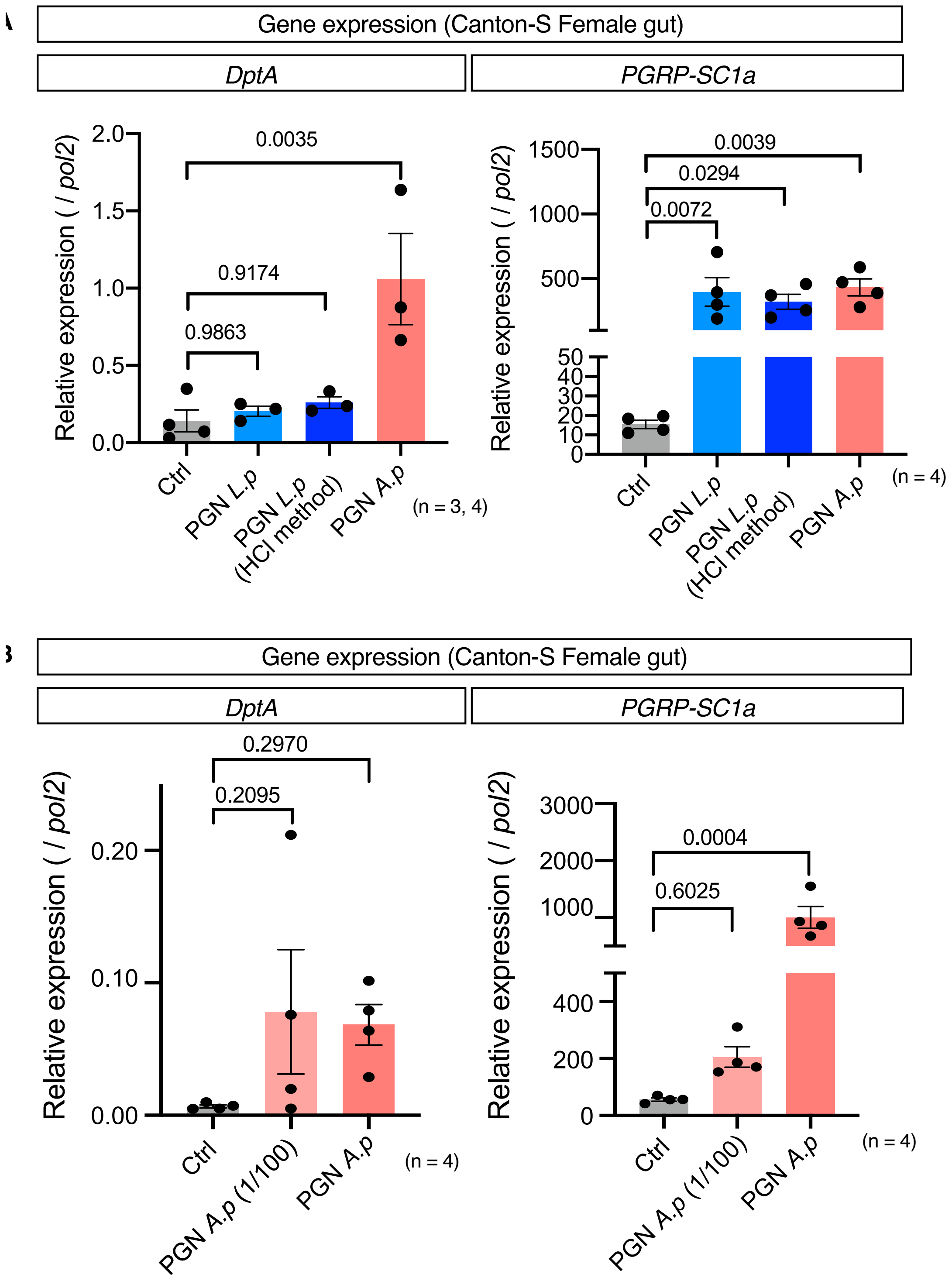

Fig 7. Feeding *L.plantarum* Lsi and *A.persici* Ai purified peptidoglycans.

(A)Quantitative RT–PCR of the genes *DptA* and *PGRP-SC1a* in female fly Canton-S guts after 24 hours of purified peptidoglycan diet with the same concentration (measured and calculated by OD_{254}). For the statistics, ANOVA with Holm-Šídák's multiple comparison

was used. (B)Quantitative RT–PCR of the genes *DptA* and *PGRP-SC1a* in female fly Canton-S guts after 24 hours of *A.persici* Ai purified peptidoglycan (1 or 100 times dilution) diet with (measured and calculated by OD_{254}). For the statistics, ANOVA with Holm-Šídák's multiple comparison was used.The control diet is the antibiotics-contained diet. Sample sizes (n) and *P* values are in each figure.

(Result) *To determine whether the differences in activity between heat-killed A. persici Ai and L. plantarum Lsi were solely attributed to the differences in PGNs rather than other components, we purified the PGNs from each bacterial species. As gram positive L. plantarum has wall teichoic acids (WTA) in the PGN layers, we also tested the effect of removing WTA on the phenotype. Strikingly, feeding with purified PGNs produced the same pattern of Imd target gene induction in the gut as BacD or HK (Fig 7A). Purified PGNs of A.persici Ai induced both DptA and PGRP-SC1a expression, while PGNs from L. plantarum Lsi induced only PGRP-SC1a expression in the gut. Removing WTA by HCl did not affect the gene expression pattern of L. plantarum Lsi at all. Furthermore, even a hundred-fold dilution of purified PGNs of A.persici Ai did not diminish the induction of DptA expression (Fig 7B). These results suggest that purified PGNs are sufficient for inducing the distinct gene expression patterns in the gut and the differences in PGN structure of two bacterial species are likely responsible for the differential stimulation of the receptors PGRP-LC/LE.*

(Discussion) *Due to the difficulty in obtaining a large amount of purified PGN, we measured only expression levels of the Imd target genes in the gut as a primary response of flies. However, given that ageing phenotypes are mediated by a "peptidoglycan"-recognition protein PGRP-LC, and caused by heat-killed A. persici Ai, we can logically attribute these phenotypes to the PGNs. Further biochemical and genetic analyses are needed to understand how specific PGN structure is recognized by the two PGRP receptors.*

2-2, Secondly, the use of bacterial-conditioned diets is a rather artificial system. It's possible to keep fly food tubes sterile during the time of an experiment, even for

lifespan study (very clean and dedicated Fly incubators and ad hoc fly and tube handling procedures under a microbiology hood should be implemented). Nevertheless, concerning the experiments presented, it will be an added value to quantify the effect of the antibiotic cocktail on the bacterial CFUs and to produce a parallel between the bacterial-conditioned diets and metabolic active bacteria.

>We thank the reviewer for this criticism that deepens discussion. We admit that our BacD could be artificial. However, given no single experiment can be perfect, it is essential to carefully consider the benefit vs drawback for an experimental system. As we have discussed in the manuscript, classical gnotobiotic analysis may sometimes produce an artefact, as we cannot control the dose of each bacterial species, which is affected by various conditions of the host. Especially, when directly comparing the effect of two or more bacterial species on host physiology, the quantity of the gut bacteria should be carefully controlled. Empirically, the gnotobiotics (i.e. monoassociation) could not always mimic physiological conditions, as animal's association with single bacterial species *per se* can be artificial. Considering that we can control bacterial quantity and quality to some extent in our BacD experiments, it may overcome some of the drawbacks of the conventional gnotobiotic experiment. Regarding "*produce a parallel between the bacterial-conditioned diets and metabolic active bacteria*", we would like to emphasize that the phenotypes observed in flies fed with BacD is reminiscent with some gnotobiotic flies we published earlier. For example, *A. persici* Ai has higher ability to induce AMPs than *L. plantarum* Lsi (Yamauchi et al., iScience, 2020, Kosakamoto et al., Cell Rep, 2020). Although not the same species, *A. aceti* FO1 decreases starvation resistance, increases paraquat resistance, induces gut AMPs, increases ISC proliferation and shortens lifespan (Obata et al., Nat Commun, 2018). These phenotypes were observed in antibiotic-free conditions. Since we have now proved that purified PGNs from *A. persici* Ai but not *L. plantarum* Lsi alone stimulates the different PGRP receptors, our data together suggested that metabolic activity has a minor effect. To further confirm this, we now added new Fig. S4, in which we tested BacD without antibiotics produced the same phenotype of BacD with antibiotics. Basically, we observed antibiotics had minimal impact on the phenotypes (note that phenotypes related to ageing was not tested due to the difficulty in maintaining the negative control germ-free in the absence of antibiotics). These data are now in the revised manuscript.

Regarding "*quantify the effect of the antibiotic cocktail on the bacterial CFUs*", our antibiotic cocktail kills all the bacteria, producing zero CFU (this is now explained in the revised manuscript).

(Methods) The dosage of antibiotics was optimised based on the viability of bacteria in BacD. The absence of live bacteria in BacD was periodically checked by plating a piece of BacD to MRS agar.

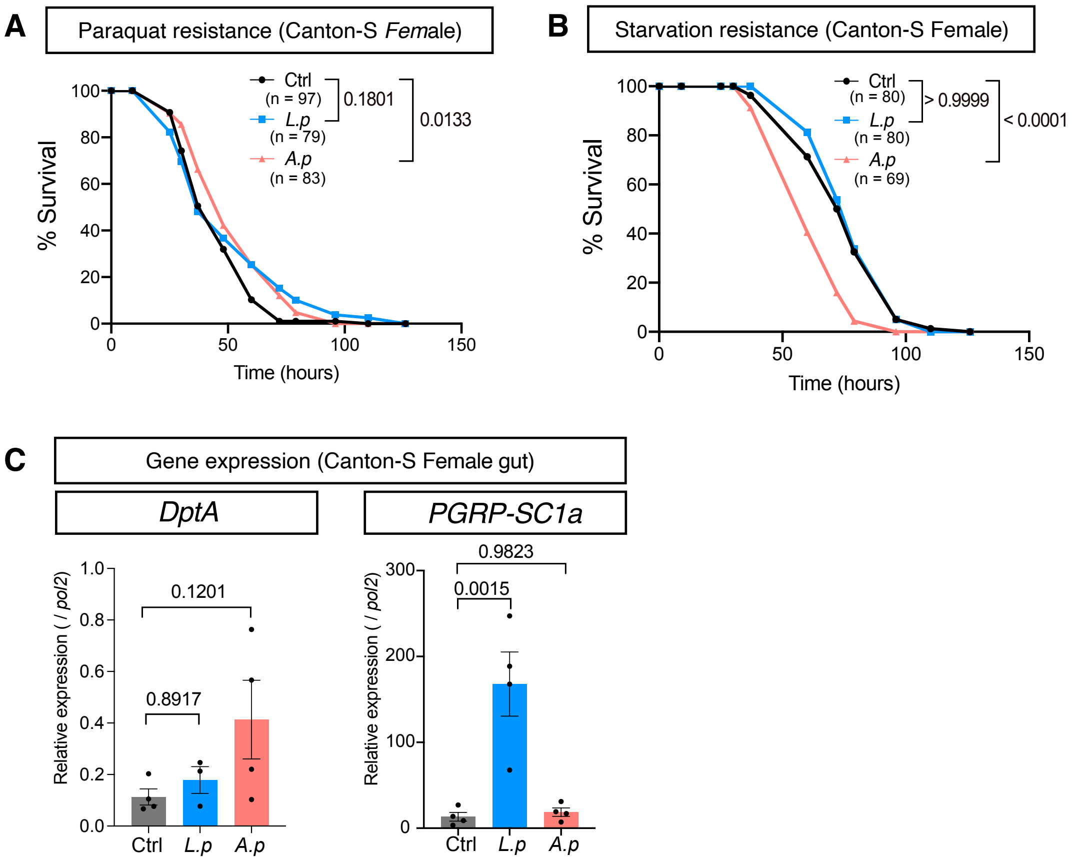

Fig S4. The effect of antibiotic cocktail on the BacD phenotypes is minimal.
(A)Survival curve of female Canton-S flies during 10 mM paraquat feeding after 5 days of a bacteria-conditioned diet (BacD) without adding the antibiotics cocktail. A log-rank test was used to compare between control (Ctrl) and BacD without antibiotics. (B) Survival curve of female Canton-S flies during starvation stress after 5 days of BacD without adding the antibiotics cocktail. A log-rank test was used to compare between control (Ctrl) and BacD without antibiotics. (C) Quantitative RT–PCR of *DptA* in female

Canton-S fly gut after 24 hours of BacD without adding the antibiotics cocktail. For the statistics, one-way ANOVA with Holm-Šídák's multiple comparison was used. The control diet has only MRS broth in place of bacterial isolates without the antibiotics cocktail. Sample sizes (n) and *P* values are in each figure.

(Result) *Since our BacDs includes the antibiotics cocktail, we checked whether side effect of antibitoics would interfere with the phenotypes caused by BacD. The differential induction of Imd target genes as well as the enhanced paraquat resistance or the reduced starvation resistance with A.persici Ai-conditioned diet was observed even in the absence of the antibiotics cocktail (Figs S4A, B, C).*

2-3, Finally, a deeper analytical study of the ”conditioned-diet” especially towards which microbial compounds and cell wall components are present is crucial to this work. A follow up on testing purified compounds is also necessary.

>We do not agree with the reviewer that the deeper analytical study is crucial for this work. Our aim of the present study is to understand the mechanism how gut microbiome shortens lifespan. BacD is a tool to achieve this. Thanks to the reviewers' comments, in the revised manuscript, we have demonstrated that the purified PGN is a key factor. If there is another microbial factor that affects a fly phenotype of our interest, then we could identify it in our future study.

2-4, In general, legends deserve to be further completed, at least, those concerning the experimental protocols.

>The legends and Methods have been modified accordingly.

Reviewer #3: In this manuscript, Onuma et al. investigated the role of microbiome, with special interests in microbiome-derived factors, on fly aging. They optimized their assay by using bacteria-conditioned diet (BacD experiement). Using this method, they found that Acetobacter (i.e. Acetobacter-conditioned diet) shortened lifespan, which is consistent with their previous finding. In addition to lifespan, they also found that Acetobacter accelerates aging phenotypes such as ISC hyperproliferation and reduced climbing ability. Acetobacter increased host resistance to paraquat and oral pathogen infection, but reduced starvation stress. They further found that Acetobacter induces IMD-dependent AMP expression in the anterior midgut via PGRP-LC, whereas Lactobacillus induces IMD-dependent PGRP-SC1a gene in the posterior midgut via PGRP-LE. Heat-killed acetobacter also shortened lifespan, but did not affect stress phenotypes. Knockdown of IMD pathway revealed that Acetobacter-activated IMD pathway is responsible for ISC hyperproliferation and early death. Taken all together, they concluded that commensal Acetobacter-derived peptidoglycan plays and important role on intestinal cell homeostasis, gut immunity, and host lifespan.

3-0, General comments.
In general, it contains some interesting aspects of the peptidoglycan-activated immune sigaling pathway in the gut (e.g. regional specificities of PGRPs and caudal regulator). However, I am not sure that their experimental condition is physiologially relevant (although I agree that BacD experiment is more technically accessbile than conventional gnotobiotic experiment). As they tested all the experiment with BacD containing a cocktail of antibiotics, this extreme and artificial condition could not reflect the real physiological roles of commensal bacteria. Therefore, although I admit that all their data are solid in their experimental settings, I am not sure whether the conclusion of this work is still valid in a physiological condition.
>We thank the reviewer for this criticism. We are glad to hear that the reviewer admit that all the data are solid. We understood that our BacD could be an artificial system, but it should be a useful tool to understand molecular mechanism of bacteria-dependent some, if not all, effects on a host. As we have discussed in the manuscript, classical gnotobiotic analysis may sometimes produce an artefact, as we cannot control the dose of each bacterial species, which is affected by various conditions of the host. Especially, when comparing two or more bacterial species, the quantity of

the gut bacteria should be carefully controlled. Empirically, the gnotobiotics (i.e. monoassociation) could not always mimic physiological conditions, as animal's association with single bacterial species *per se* can be artificial. Considering that we can control bacterial quantity and quality to some extent in our BacD experiments, it may overcome some of the drawbacks of the conventional gnotobiotic experiment. We would like to emphasize that the phenotypes observed in flies fed with BacD is consistent with some gnotobiotic flies we published earlier. For example, *A. persici* Ai has higher ability to induce AMPs than *L. plantarum* Lsi (Yamauchi et al., iScience, 2020, Kosakamoto et al., Cell Rep, 2020). Although not the same species, *A. aceti* FO1 decreases starvation resistance, increases paraquat resistance, induces gut AMPs, increases ISC proliferation and shortens lifespan (Obata et al., Nat Commun, 2018). If we take all these findings into consideration, it is difficult to conclude our BacD experiments just produce something artificial.

Specific comments.

3-1) One of the problems in BacD experiment is that they use a single condition with relatively high number of bacteria. It may be helpful to see the result (e.g. survival) by using different number of bacteria (i.e. dose-dependent experiments).

> First, we would like to mention that our BacD does not necessarily have too much bacteria. We determined the dose of inoculation for BacD by referring typical bacterial number in a standard diet cultured with wild type flies. For example, three days after rearing Canton S male flies in a vial produced $5.54*10^4$ CFU/mg food. This is comparable with that in BacD (Fig. 1B, ~$19.1*10^4$ (*L.p*) or $5.14*10^4$ (*A.p*) CFU/mg food). We revised manuscript accordingly.

(Results) *Incubation of the diet at 25 °C for 24 hours allowed the bacterial species to proliferate approximately one hundred-fold (Fig 1A and 1B), reaching $19.1*10^4$ (L. plantarum) or $5.14*10^4$ (A. persici) CFU/mg food that is comparable to the typical bacterial numbers in the standard diet with wild type flies (~10^{4-5} CFU/mg food in case of 1 week old Canton-S male flies) in our laboratory.*

(Methods) *We chose OD_{600}=0.2 or 0.4 as it produces comparable level of CFU in the standard diet after keeping wild type flies for three days.*

Regarding "*It may be helpful to see the result (e.g. survival) by using different number of bacteria (i.e. dose-dependent experiments)*", we agree that testing dose-dependency is informative. To better control the dose of bacterial effect, we prepared heat-killed bacteria with various doses. New Figs S5D, E shows that HK *A. persici* Ai induced *DptA* at the lowest dose (OD_{600}=0.4) while *L. plantarum* Lsi could not do so even at the highest dose (OD_{600}=40) (Fig S5D). In contrast, *L. plantarum* Lsi induced *PGRP-SC1a* expression significantly at the middle dose (OD_{600}=4), while *A. persici* Ai boosted it only at the highest dose (Fig. S5D, E). These data highlight the differential activity of stimulation of PGRP receptors by each bacterial species.

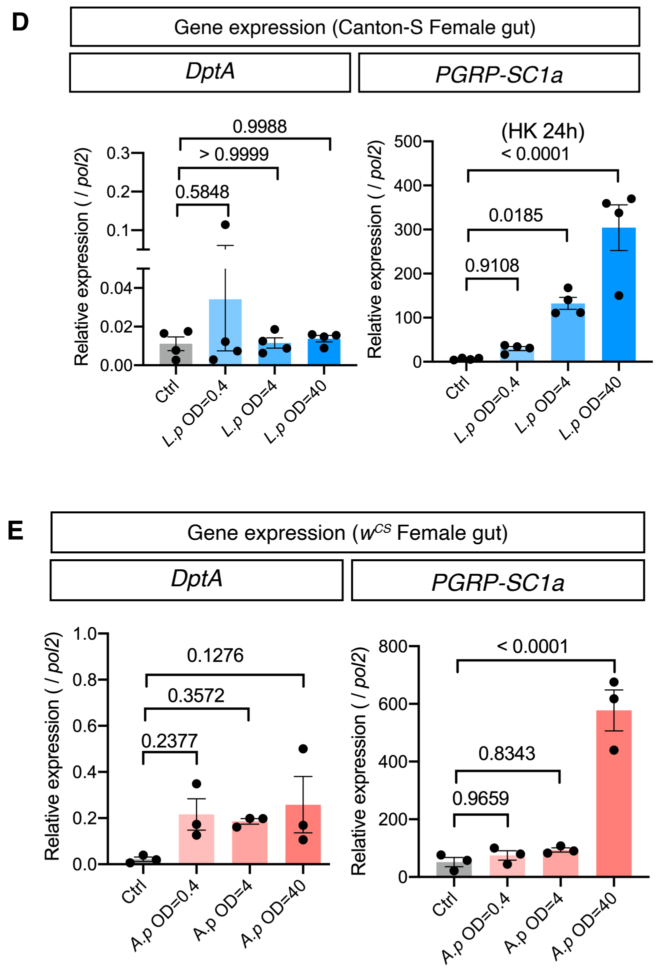

Fig S5D, E. Quantitative RT–PCR of *DptA* and *PGRP-SC1a* after 24 hours of several dilutions of HK with either *L.plantarum* (D) or *A.persici* (E). For the statistics, one-way ANOVA with Holm-Šídák's multiple comparison was used.

(Results) *HK A. persici Ai induced DptA even at the lowest dose (OD_{600}=0.4) while L. plantarum Lsi could not do so even at the highest (OD_{600}=40) (Figs S5D, E). In contrast, L. plantarum Lsi induced PGRP-SC1a expression significantly at the middle dose (OD_{600}=4), while A. persici Ai boosted it only at the highest dose (Fig. S5D, E). These data highlight the differential stimulation potential of PGRP receptors by each bacterial species.*

3-2) It is often observed that antibiotics slightly affect the fitness of germ-free Drosophila. Do they test the effect of antibiotics?

> As stated in our comment 2-2, the phenotypes observed in flies fed with BacD is reminiscent with gnotobiotic flies, suggesting that antibiotics and/or bioactivity of bacterial species *per se* did not affect the outcome of experiments. We now added new Fig. S4, in which we tested BacD without antibiotics produced the same phenotype of BacD with antibiotics. Basically, we observed antibiotics had minimal impact on the phenotypes (note that phenotypes related to ageing was not tested due to the difficulty in maintaining the negative control germ-free in the absence of antibiotics).

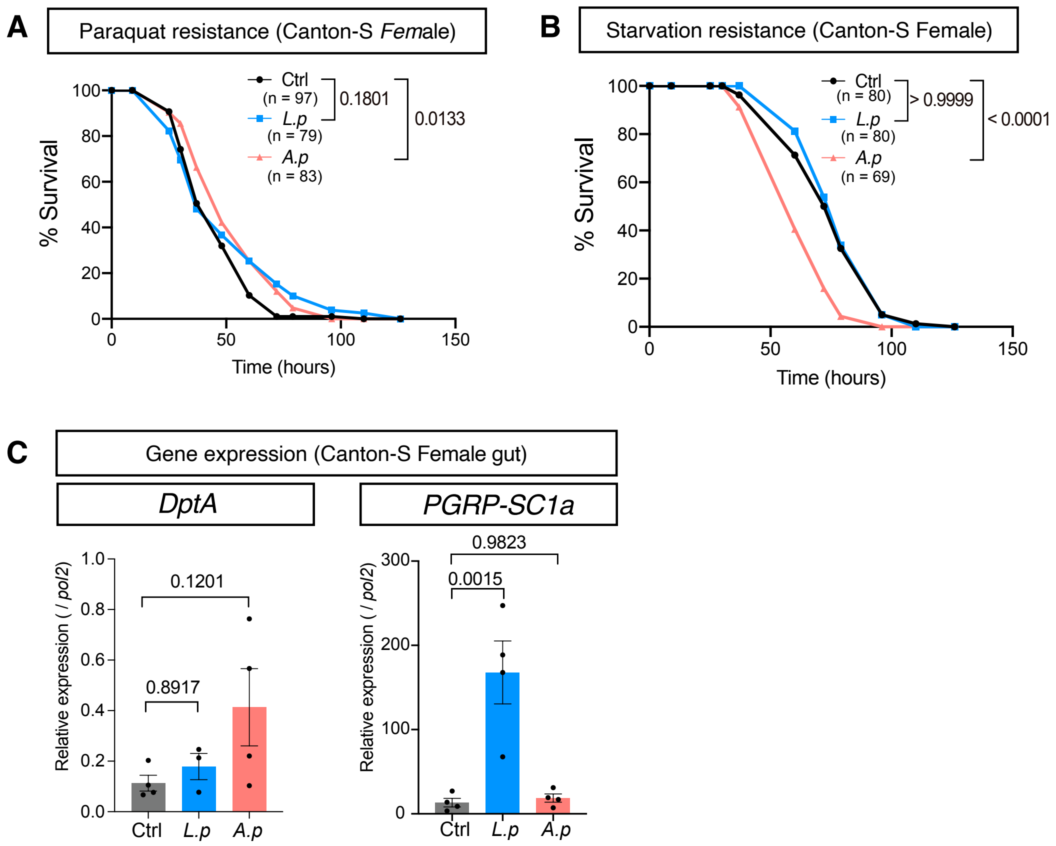

Fig S4. The effect of antibiotic cocktail on the BacD phenotypes is minimal.

(A)Survival curve of female Canton-S flies during 10 mM paraquat feeding after 5 days of a bacteria-conditioned diet (BacD) without adding the antibiotics cocktail. A log-rank test was used to compare between control (Ctrl) and BacD without antibiotics. (B) Survival curve of female Canton-S flies during starvation stress after 5 days of BacD without adding the antibiotics cocktail. A log-rank test was used to compare between control (Ctrl) and BacD without antibiotics. (C) Quantitative RT–PCR of *DptA* in female Canton-S fly gut after 24 hours of BacD without adding the antibiotics cocktail. For the statistics, one-way ANOVA with Holm-Šídák's multiple comparison was used. The control diet has only MRS broth in place of bacterial isolates without the antibiotics cocktail. Sample sizes (n) and *P* values are in each figure.

(Result) *Since our BacDs includes the antibiotics cocktail, we checked whether side effect of antibitoics would interfere with the phenotypes caused by BacD. The*

differential induction of Imd target genes as well as the enhanced paraquat resistance or the reduced starvation resistance with A.persici Ai-conditioned diet was observed even in the absence of the antibiotics cocktail (Figs S4A, B, C).

3-3) It is unclear whether life-shortening effect is a general asepct of acetobacter. They should test different species of acetobacter (preferentially originated from other laboratories). Is there any difference between BacD experiement and gnotobiotic experiement?

> The corresponding author had already reported that *Acetobacter* aceti FO1 and *Acetobacter* sp. FO3 (Obata et al., Nat Commun, 2018) shortens fly lifespan in his previous laboratory in the UK, increasing generality of (live) *Acetobacter*'s effect. This lifespan-shortening activity of *Acetobacter* species were also observed in Japan where I had launched my own group, using newly isolated strain *A. persici* Ai. We have also reported that live *Gluconobacter* sp. Gdi (Kosakamoto et al., Cell Rep, 2020) shortens fly lifespan. As *Gluconobacter* is an *Acetobacteraceae*, we assume that wide variety of *Acetobacteraceae* family bacteria can shorten fly lifespan. In this study, we showed BacD with *A. persici* Ai or *Gluconobacter* sp. Gdi shortens lifespan. Therefore, the life-shortening effect of *Acetobacteraceae* is commonly observed in both gnotobiotic and BacD experiments.

3-4) Why and how does acetobacter affect host resistance against oxidative stress and starvation? What about the IMD knockdown animals (in ISC/EB as well as in EB/EC) under BacD experiment or under gnotobiotic experiement?

> In the new Fig S6F, we found that knockdown of PGRP-LC in the enterocytes and enteroblasts (using *5966*^{GS}) suppressed the enhanced resistance to paraquat, suggesting that PGRP-LC-dependent Imd activation in the gut enhances paraquat resistance. The mechanism how the intestinal Imd activity changes paraquat resistance is not known yet. Increasing epithelial tolerance by *A. persici* Ai would be key mechanism, but further analysis is necessary. We added this figure in the revised manuscript. Since we assumed that the drop of starvation resistance by *A. persici* Ai-

conditioned diet was a metabolic phenotype rather than one related to gut epithelia, we did not test this.

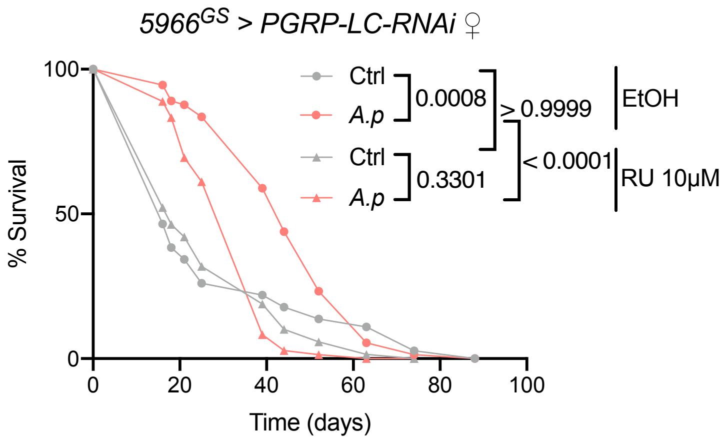

Fig S6F, Resistance to 10mM paraquat of *5966*^{GS}*>PGRP-LC-RNAi* flies after 5 days of BacD (with EtOH or RU486) were measured. Survival curve of female flies during 10 mM paraquat feeding after 5 days of BacD and ethanol or RU 10μM. A log-rank test was used to compare between control (Ctrl) and BacD with ethanol or 10μM RU.

(Result) *On the other hand, knockdown of PGRP-LC by 5966*^{GS} *suppressed the enhanced resistance to paraquat by A. persici Ai-conditioned diet, suggesting that PGRP-LC-dependent Imd activation in the gut improves paraquat resistance (Fig S6F).*

3-5) It is interesting to see the effect of Acetobacter on host overexpressing PGRP-SC in the posterior midgut.

>We thank the reviewer for this interesting suggestion. Unfortunately, we are not aware of any posteior midgut-specific gal4 drivers available. Instead, we checked whether PGRP-SC2 overexpression in the entire gut (using 5966^{GS}) influenced the Imd activation. Unfortunately, in this experimental setting, we could not find any suppression of AMPs induction by *A. persici* Ai, implying it was not effective to modulate PGRP-LC-dependent phenotypes (Fig. R2). We tested only PGRP-SC2 not PGRP-SC1a/b as it is the only UAS-PGRP-SC stock currently available for us. Further gain- or loss-of-function genetic experiments, preferably by the posterior midgut-

specific drivers, are necessary for concluding it. We believe it is beyond the scope of the present study.

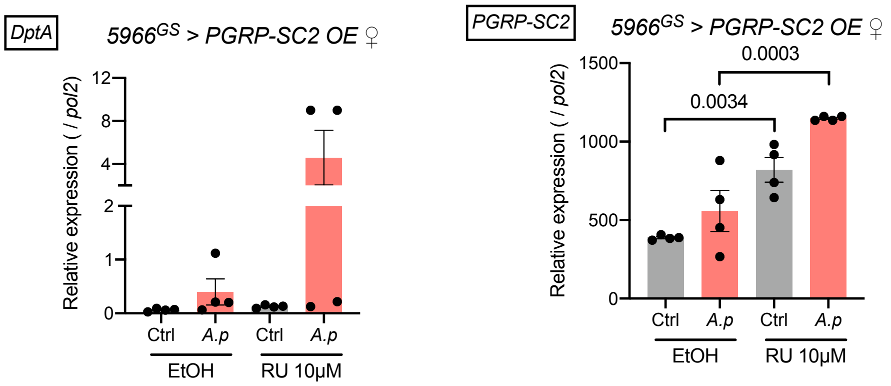

Fig R2, Gut gene expression of female *5966^{GS}>PGRP-SC2* after 24 hours of HK.

3-6) It is unclear why Lactobacillus does not activate the IMD pathway in the anterior midgut. They should use peptidoglycan (partially purified from Lactobacillus) to see whether it could activate the IMD pathway in the anterior midgut.

3-7) Why do peptidoglycans of different bacteria use different receptors? Is it due to the different structure? What happens if they use partially-purified peptidoglycans from acetobacter and lactobacillus?

> This is indeed key question for the complete understanding. As we stated in our comments 1-10 and 2-1, we succeeded to purify peptidoglycans from both bacterial species and to remove teichoic acids from gram-positive *L. plantarum* Lsi. We found that the purified PGNs phenocopied BacD (new Fig. 7A,B). These data and relevant text are now in the revised manuscript.

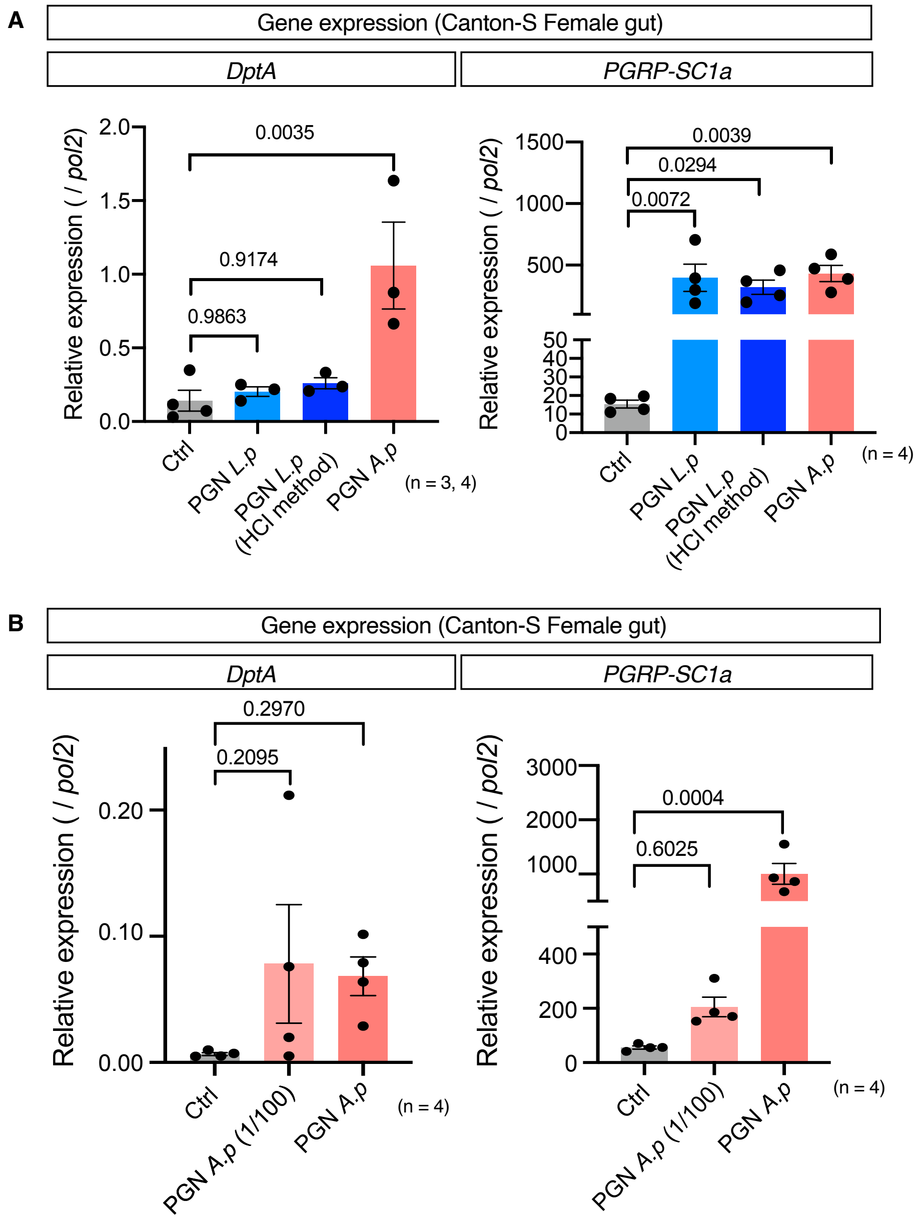

Fig 7. Feeding *L.plantarum* Lsi and *A.persici* Ai purified peptidoglycans.

(A)Quantitative RT–PCR of the genes *DptA* and *PGRP-SC1a* in female fly Canton-S guts after 24 hours of purified peptidoglycan diet with the same concentration (measured and calculated by OD_{254}). For the statistics, ANOVA with Holm-Šídák's multiple comparison was used. (B)Quantitative RT–PCR of the genes *DptA* and *PGRP-SC1a* in female fly Canton-S guts after 24 hours of *A.persici* Ai purified peptidoglycan (1 or 100 times dilution) diet with (measured and calculated by OD_{254}). For the statistics, ANOVA with Holm-Šídák's multiple comparison was used.The control diet is the antibiotics-contained

diet. Sample sizes (n) and *P* values are in each figure.

(Result) *To determine whether the differences in activity between heat-killed A. persici Ai and L. plantarum Lsi were solely attributed to the differences in PGNs rather than other components, we purified the PGNs from each bacterial species. As gram positive L. plantarum has wall teichoic acids (WTA) in the PGN layers, we also tested the effect of removing WTA on the phenotype. Strikingly, feeding with purified PGNs produced the same pattern of Imd target gene induction in the gut as BacD or HK (Fig 7A). Purified PGNs of A.persici Ai induced both DptA and PGRP-SC1a expression, while PGNs from L. plantarum Lsi induced only PGRP-SC1a expression in the gut. Removing WTA by HCl did not affect the gene expression pattern of L. plantarum Lsi at all. Furthermore, even a hundred-fold dilution of purified PGNs of A.persici Ai did not diminish the induction of DptA expression (Fig 7B). These results suggest that purified PGNs are sufficient for inducing the distinct gene expression patterns in the gut and the differences in PGN structure of two bacterial species are likely responsible for the differential stimulation of the receptors PGRP-LC/LE.*

(Discussion) *Due to the difficulty in obtaining a large amount of purified PGN, we measured only expression levels of the Imd target genes in the gut as a primary response of flies. However, given that ageing phenotypes are mediated by a "peptidoglycan"-recognition protein PGRP-LC, and caused by heat-killed A. persici Ai, we can logically attribute these phenotypes to the PGNs. Further biochemical and genetic analyses are needed to understand how specific PGN structure is recognized by the two PGRP receptors.*

Regarding the comment 3-6) "*why Lactobacillus does not activate the IMD pathway in the anterior midgut*", we showed that PGNs from *L. plantarum* Lsi with or without teichoic acids could not upregulate AMPs, suggesting that they still fail to activate Imd pathway from the anterior midgut. This supports the hypothesis that PGN structure (presumably modification) from *L. plantarum* Lsi is not capable of stimulating PGRPs in

the anterior midgut, but it is converted to an "active" form in the posterior midgut. We added this explanation in the revised manuscript.

(Results) *Why L. plantarum Lsi does not activate the Imd pathway through PGRP-LE in the anterior gut is not clear; however, one can assume that the L. plantarum Lsi PGNs may be converted to "active" form (through modification, etc.) that allows it to be recognized by the receptor in the posterior region.*

3-8) It is known that IMD pathway is not involved in ISC turnover (e.g. Buchon et al.). How does IMD pathway in ISC/EB affect ISC proliferation? Is enterocyte-specific IMD pathway involved in ISC turnover? In the same context, is ISC/EB-specific IMD pathway involved in lifespan?

> We thank the reviewer for this comment that strengthen the mechanistic understanding of ageing promotion by *A. persici* PGNs. It has been shown that Imd pathway components including PGRP-LC are enriched in ISCs and Imd activation promotes the ISC proliferation autonomously (Petkau et al., Cell Rep, 2017, Shin et al., Stem Cell Rep, 2022). Consistent with these reports, our data suggested that *Acetobacter*'s PGN stimulates PGRP-LC-dependent Imd pathway directly in stem cells and promote proliferation.

Nevertheless, it is possible that Imd activation in enterocytes can non-cell autonomously promotes ISC proliferation. To test this, during revision, we quantified ISC proliferation in *NP1^{ts}>PGRP-LC-RNAi* flies. As new Fig. S6A showed, *A. persici* Ai failed to promote ISC proliferation in *NP1^{ts}>PGRP-LC-RNAi*, suggesting that PGRP-LC in enterocytes was also necessary for ISC proliferation. Furthermore, knockdown of PGRP-LC using *esg^{ts}* mitigated lifespan reduction by HK *A. persici* Ai from a 19% decrease in the median lifespan to a 7% decrease (Fig 8E), suggesting that PGRP-LC in progenitor cells plays a role in lifespan. Together, stimulation of PGRP-LC in both cell types in the gut contribute to gut ageing and organismal lifespan.

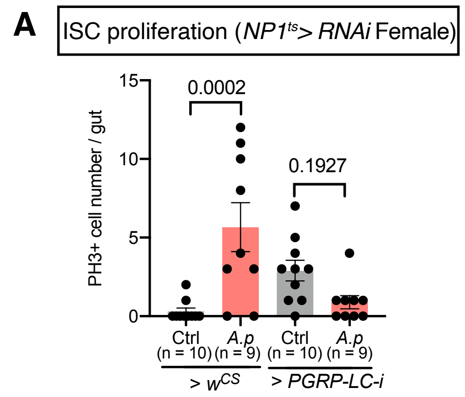

Fig S6A Phospho-histone H3-positive cell numbers in the midgut of *NP1ts > PGRP-LC-RNAi* flies after 38days of HK. Flies were reared at 30 °C during their adult period. For the statistics, one-way ANOVA with Holm-Šídák's multiple comparison was used.

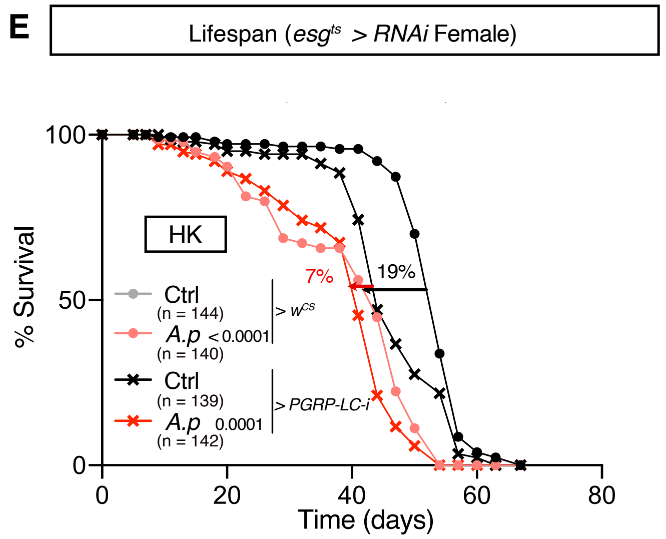

Fig 8E Lifespan of female *esgts > PGRP-LC-RNAi* flies with HK. Flies were reared at 30 °C during their adult period. A log-rank test was used to compare between control (Ctrl) and HK in each genotype. The 50% survival rate is compared.

(Results) *Interestingly, PGRP-LC-knock down in enterocytes by NP1ts also attenuated the ISC proliferation (Fig. S5A), suggesting that enterocytes' PGRP-LC also contributed to promotion of age-related ISC proliferation by A. persici Ai.*

(Results) *Since the increase of ISC proliferation caused by A.persici Ai was mitigated by knockdown of PGRP-LC in progenitor cells, we asked whether this also affected lifespan. Knockdown of PGRP-LC using esg^{ts} mitigated lifespan reduction by HK A. persici Ai from a 19% decrease in the median lifespan to a 7% decrease (Fig 8E).*

3-9) In the text, they stated that "These metabolite alterations suggested that BacD would be suitable for understanding how bacteria and bacterial products influence their hosts and, thereby, possibly recapitulate the phenotypes seen in flies with a monoassociation" However, I think there are big differences between BacD and gnotobiotic experiment.

>We thank the reviewer for this critical comment. This could have been an overstatement. We weakened this sentence as follows.

(Results) *These metabolite alterations suggested that BacD can be utilised for understanding how bacteria and bacterial products influence their hosts.*
